# A Rare Presentation of Erosive Gastritis Caused by Helicobacter heilmannii Infection

**DOI:** 10.7759/cureus.86876

**Published:** 2025-06-27

**Authors:** Adarsh Jha, Divij K Jha, Amey U Joshi, Samiksha Pandey, Fariah K Ahmad

**Affiliations:** 1 Internal Medicine, Michigan State University, Lansing, USA; 2 Gastroenterology and Hepatology, Oakland University William Beaumont Hospital, Royal Oak, USA; 3 Gastroenterology, University of Michigan Health-Sparrow, Lansing, USA

**Keywords:** erosive gastritis, helicobacter heilmannii, helicobacter pylori, malt lymphoma, peptic ulcer

## Abstract

*Helicobacter heilmannii* (*H. heilmannii*) is an uncommon gastric pathogen increasingly recognized for its role in gastrointestinal diseases. Unlike *Helicobacter pylori* (*H. pylori*), *H. heilmannii* is rarely detected and often overlooked. It is typically associated with milder pathological features, including less neutrophilic activity, reduced mononuclear infiltration, and endoscopic signs of chronic gastritis without erosions or ulcers.

We report a case of a 71-year-old female patient with a history of invasive breast carcinoma who presented with dysphagia, nausea, and dyspepsia following chemoradiotherapy. Initial endoscopy revealed esophageal strictures and erosive gastritis, with biopsies negative for *H. pylori*. Persistent symptoms prompted repeat endoscopic evaluation, which demonstrated *H. heilmannii* on histology. The patient responded well to bismuth quadruple therapy, with complete symptom resolution and eradication of the infection.

This case underscores the need to consider *H. heilmannii* as an uncommon but potential cause of unexplained erosive gastritis or peptic ulcer disease, given its typically milder histopathologic features, such as reduced neutrophilic and mononuclear infiltration and subtle endoscopic findings, which may contribute to underdiagnosis. Prompt recognition and treatment can lead to excellent clinical outcomes and may reduce the risk of long-term complications such as mucosa-associated lymphoid tissue (MALT) lymphoma.

## Introduction

*Helicobacter heilmannii* (*H. heilmannii*) is an infrequently encountered gastric *Helicobacter* species compared to the more prevalent *Helicobacter pylori* (*H. pylori*) but has been implicated in several clinically important gastrointestinal conditions, including chronic gastritis, peptic ulcer disease, and an elevated risk of gastric carcinoma and mucosa-associated lymphoid tissue (MALT) lymphoma [1].

## Case presentation

We present a case of a 71-year-old female patient with a past medical history of invasive ductal and lobular carcinoma of the right breast. She had been treated with surgery followed by chemoradiotherapy. Approximately 16 weeks after completing treatment, she developed progressive solid-food dysphagia accompanied by nausea and epigastric discomfort. On examination, her vital signs were stable, and there were no abdominal masses or organomegaly. Initial laboratory tests were unremarkable.

Due to her history of thoracic radiation, esophagogastroduodenoscopy (EGD) was performed to evaluate dysphagia and dyspeptic symptoms. EGD revealed radiation-induced esophageal changes, including areas of stricture in the upper esophagus, as well as diffuse erosive gastritis in the stomach. Multiple gastric biopsies were obtained. Rapid urease testing and histopathologic examination of these initial gastric biopsies were negative for *H. pylori*. The patient underwent endoscopic dilatation of the esophageal strictures and was managed with proton pump inhibitor therapy. Despite this, her symptoms of dyspepsia and nausea persisted over the following two months.

**Figure 1:**
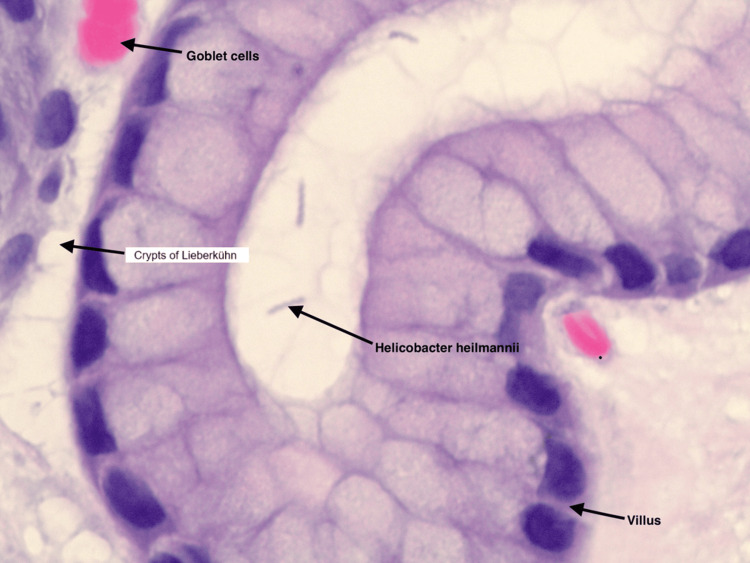
Duodenal biopsy showing Helicobacter heilmannii organisms with minimal inflammation (H&E stain) H&E: hematoxylin and eosin

**Figure 2:**
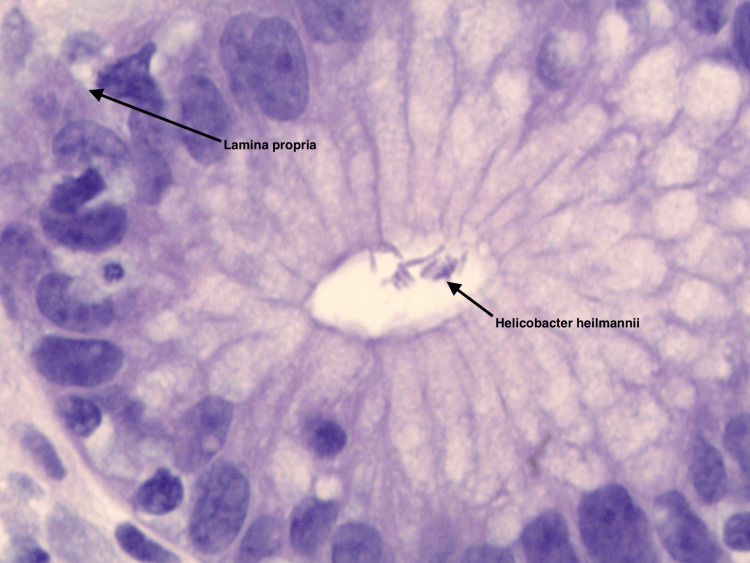
Hematoxylin and eosin-stained section of gastric biopsy showing numerous tightly coiled, spiral-shaped organisms morphologically consistent with Helicobacter heilmannii, accompanied by minimal inflammatory infiltrate within the lamina propria

A repeat EGD was then performed to reassess her persistent symptoms. The gastric mucosa still showed erythema and erosions. Biopsies from the atrium and body were obtained for histological evaluation. As shown in Figures 1, 2, respectively, numerous tightly coiled, spiral-shaped organisms morphologically consistent with *H. heilmannii*, with only minimal inflammatory cell infiltration, were observed [1]. This contrasts with *H. pylori*, where the presence of marked neutrophilic and mononuclear infiltration in the gastric mucosa typically heightens clinical and histopathological suspicion for *Helicobacter* infection, thereby facilitating its detection.

After diagnosis, the patient was treated with a two-week course of bismuth-based quadruple therapy (bismuth, proton pump inhibitor, metronidazole, and tetracycline) and tolerated the regimen well. On follow-up, the patient reported complete resolution of her dysphagia, nausea, and dyspepsia. However, recurrence of dysphasia led to a repeat EGD with biopsy and dilation four weeks after completing therapy, which showed no evidence of *Helicobacter* organisms. The patient remained asymptomatic, and no further complications were observed on subsequent evaluations.

## Discussion

This case underscores the need to consider *H. heilmannii* as an uncommon but potential cause of unexplained erosive gastritis or peptic ulcer disease, given its typically milder histopathologic features, such as reduced neutrophilic and mononuclear infiltration and subtle endoscopic findings, which may contribute to underdiagnosis. Prompt recognition and treatment can lead to excellent clinical outcomes and may reduce the risk of long-term complications such as MALT lymphoma [1-3].

*H. heilmannii* is a zoonotic pathogen associated with chronic gastritis, peptic ulcer disease, and gastric neoplasms. *H. heilmannii* tends to cause milder gastritis histopathologically than *H. pylori*, with reduced neutrophil activity and less mononuclear cell infiltration [2,3]. Endoscopic findings in *H. heilmannii* infection may also be subtle, often lacking the prominent ulcers or nodularity associated with *H. pylori* gastritis, which can contribute to underdiagnosis. In this case, the initial endoscopy showed erosive gastritis, but standard testing did not immediately identify an infectious cause. This could be due to the organism’s uncommon nature and the relatively mild inflammatory response it induces. *H. heilmannii* is typically acquired zoonotically through close contact with animals such as cats, dogs, or pigs [3]. The patient in this case had a pet dog, which is a possible source of exposure, underscoring the importance of obtaining a thorough history of animal contact when evaluating unexplained gastritis.

Despite usually mild clinical and pathologic features, *H. heilmannii* infection can occasionally present as severe gastrointestinal disease. Case reports have described acute erosive gastritis and peptic ulcer disease attributable to *H. heilmannii* infection [4-6]. Yamamoto et al. described an asymptomatic 71-year-old patient who was found to have erosive gastritis with *H. heilmannii* identified on cytology. Another case documented acute gastric mucosal lesions associated with *H. heilmannii* on endoscopy [4,5]. More recently, a rare instance of peptic ulcer disease due to non-*H. pylori* species was reported in a patient with acute upper gastrointestinal bleeding [6]. Collectively, these case reports, including our case, demonstrate that *H. heilmannii* can occasionally lead to significant gastric mucosal damage and clinical symptoms that mimic more common etiologies of peptic ulcer disease.

In addition to acute manifestations, the pathogen also poses long-term risks. Chronic infection with the organism has been linked to serious complications, including gastric neoplasms such as carcinoma and MALT lymphoma [1]. Notably, there is evidence that treating *H. heilmannii* infection can lead to regression of associated malignancy. Okamura et al. reported a case of primary gastric MALT lymphoma that achieved complete remission after eradication of an *H. heilmannii* infection [7]. Given the association of *H. heilmannii* with MALT lymphoma and possibly gastric cancer, prompt identification and eradication of this organism are advisable even in the absence of severe symptoms. While no specific guidelines exist for *H. heilmannii*, standard *H. pylori* eradication regimens appear effective. In our case, bismuth-based quadruple therapy led to complete symptom resolution and presumed cure.

This case highlights the importance of considering *H. heilmannii* infection in patients with unexplained erosive gastritis or refractory dyspepsia, especially when *H. pylori* testing is negative. Increased awareness of this rare pathogen can facilitate appropriate diagnostic measures, such as meticulous histological review or molecular testing, and timely initiation of eradication therapy. Early recognition and treatment of *H. heilmannii* may lead to excellent outcomes and help prevent potential long-term complications.

## Conclusions

In conclusion, *H. heilmannii* is a rare but clinically significant cause of gastritis that can manifest with severe erosive disease. The organism should be considered in the differential diagnosis of patients presenting with persistent gastritis or peptic ulcer symptoms when *H. pylori* and other common etiologies have been excluded. Careful histopathologic evaluation of gastric biopsies is essential for diagnosis, as routine tests may overlook *H. heilmannii*. Timely diagnosis and treatment of *H. heilmannii* infection can result in symptom resolution and may prevent serious complications such as gastric MALT lymphoma.
